# Silencing of a NRDE-3 transgene in *C. elegans* germ cells and early embryos is mediated by the RNAi pathway

**DOI:** 10.17912/micropub.biology.001308

**Published:** 2024-08-16

**Authors:** Shihui Chen, Carolyn M Phillips

**Affiliations:** 1 Biological Sciences, University of Southern California, Los Angeles, CA, United States

## Abstract

Transgenes are highly susceptible to gene silencing in the *C. elegans* germline. Here, we examine the expression of the nuclear Argonaute protein NRDE-3 , comparing two GFP::NRDE-3 strains, one constructed by bombardment and one by CRISPR. We found that the GFP::NRDE-3 strain constructed by bombardment displays transgene silencing in germline and early embryos and that NRDE-3 expression can be restored in a *rde-3* mutant, which disrupts the RNAi pathway. This finding reveals that NRDE-3 is not a soma-specific Argonaute protein and is, in fact, expressed in the proximal germline and early embryos.

**Figure 1. Localization of two GFP::NRDE-3 constructs across developmental stages:**
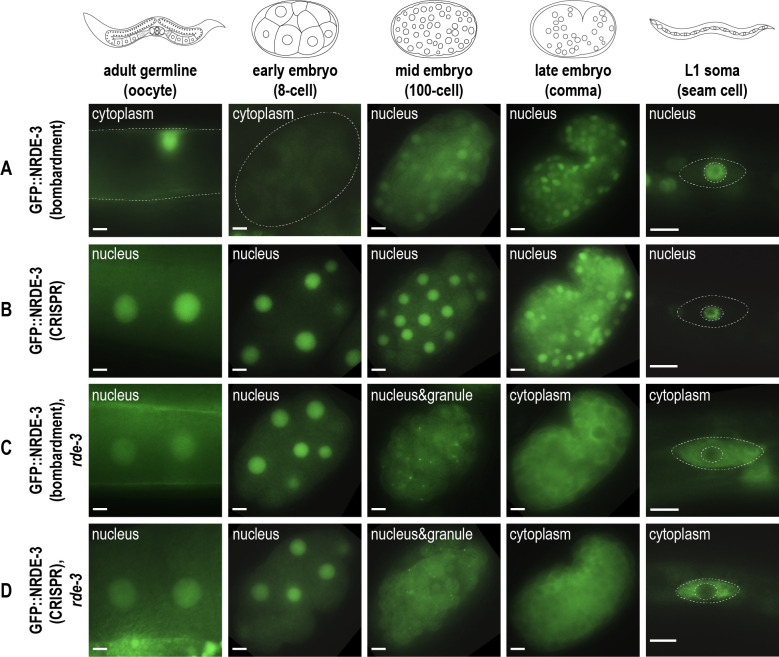
A,B: Representative live images of GFP::NRDE-3 strains generated by bomardment (A) and CRISPR (B) at different developmental stages. NRDE-3 localizes to the nuclei of oogenic germ cells, embryos of all developmental stages, and somatic seam cells in L1 larvae in the CRISPR-tagged strain (B), but is not expressed in the oocytes and early embryos in the strain generated by bomardment (A). C,D: Representative live images of GFP::NRDE-3 strains generated by bomardment (C) and CRISPR (D) at different developmental stages, in the *rde-3* mutant background. NRDE-3 localizes to the nuclei of oogenic germ cells and early embryos, to both nuclei and cytoplasmic granules in mid-stage embryos, and to the cytoplasm of late embryos and somatic seam cells in L1 larvae. Scale bars, 5 µm. For each stage, at least 10 individual embryos or animals were observed. Dotted white lines trace the edge of the proximal germline, the perimeter of the embryo, or the perimeter of the seam cell and nucleus.

## Description

Gene editing techniques are widely used in eukaryotic model systems to tag, mutate, or delete genes for experimental analysis. In *C. elegans ,* foreign DNA can be introduced by microinjection into the syncytial gonad, where it assembles via homologous recombination into extrachromosomal DNA arrays (Mello et al. 1991) . However, high-copy, extrachromosomal arrays can lead to overexpression and are not faithfully transmitted from parent to offspring (Fire and Waterston 1989) . Furthermore, in the *C. elegans* germline, high-copy arrays are treated as heterochromatic and silenced (Kelly et al. 1997, 2002) , leading to reduced expression of the transgene, as well as any endogenous, germline-expressed genes with the same sequence - a phenomenon known as co-suppression (Dernburg et al. 2000; Ketting and Plasterk 2000) . A number of alternative transgenic techniques were subsequently developed with the aim of achieving consistent germline gene expression. Microparticle bombardment (biolistic transformation) can be used to produce low-copy transgene insertions in the genome (Praitis et al. 2001) , and transposon-based methods have been developed to achieve single-copy transgene insertion by homologous recombination - either at the endogenous locus using a nearby Mos1 transposon insertion (MosTIC) (Robert and Bessereau 2007) or using Mos1 excision a defined landing site (MosSCI) (Frøkjær-Jensen et al. 2008). With the advent of the CRISPR technique (Ran et al. 2013; Doudna and Charpentier 2014; Friedland et al. 2013) , modification of the endogenous locus has become more straightforward, leading to fewer concerns about silencing and expression that more faithfully reflects the true expression of the gene. Despite this advancement, many currently used *C. elegans* strains were built with a variety of transgenic techniques. Therefore, it is important to understand the nature of the fluorescent reporters that we are using and to assess whether they are a true reflection of native protein expression.

The nuclear Argonaute protein NRDE-3 has been reported to be expressed in the nucleus of most somatic cells starting around the ~80-cell stage of embryonic development (Guang et al. 2008) . Thus, we were surprised to see, in a recent publication that CRISPR-tagged all *C. elegans* Argonaute proteins, expression of NRDE-3 in oocytes and early embryos (Seroussi et al. 2023, 2022) . Because we are interested in a number of aspects of NRDE-3 small RNA loading and function, we obtained both the original, presumably low-copy GFP::NRDE-3 transgenic line, which was generated by bombardment (Guang et al. 2008) , and the endogenously CRISPR-tagged GFP::NRDE-3 (Seroussi et al. 2023) . Consistent with previous publications, GFP::NRDE-3 (bombardment) localizes to the nuclei of somatic cells beginning around 100-cell stage of embryogenesis (Fig.1A). No expression can be detected in germ cells or early embryos. In contrast, GFP::NRDE-3 (CRISPR) is visible in nuclei beginning in late pachytene of the germline and continues to be expressed in oocytes and in all stages of embryogenesis (Fig.1B). This discrepancy in expression between the transgenic strain constructed by bombardment and the CRISPR strain suggests that GFP::NRDE-3 (bombardment) may be silenced in the germline and early embryos.

Transgene silencing and the related phenomenon of co-suppression are intimately linked with the RNAi pathway. Specifically, components of the *mutator* 22G-RNA amplification pathway (i.e. MUT-7 , MUT-16 , RDE-2 , and RDE-3/ MUT-2 ) are required for co-suppression, while the upstream, primary Argonaute protein, RDE-1 , is not (Dernburg et al. 2000; Ketting and Plasterk 2000; Vastenhouw et al. 2003) . To determine whether RNA silencing is leading to this discrepancy in expression between GFP::NRDE-3 (bombardment) and GFP::NRDE-3 (CRISPR), we introduced a mutation in *rde-3* (also known as *mut-2* ) into both GFP::NRDE-3 strains. RDE-3 encodes for a poly-UG polymerase that is critical for the production of secondary WAGO class 22G-RNAs (Chen et al. 2005; Phillips et al. 2014; Shukla et al. 2020) . Thus, it is important to note that, while loss of RDE-3 can desilence transgenes, it also results in the depletion of the WAGO class 22G-RNAs, including the ERGO-dependent 22G-RNAs to which NRDE-3 is reported to bind (Guang et al. 2008; Phillips et al. 2014; Seroussi et al. 2023) . Localization of NRDE-3 to the nucleus is dependent on small RNA binding (Guang et al. 2008) ; therefore, following introduction of the *rde-3* mutation, GFP::NRDE-3 is redistributed from the nucleus to the cytoplasm in mid/late embryogenesis and somatic cells in both strains (Fig 1C-D). We also observed that GFP::NRDE-3 in both strains localizes to cytoplasmic granules during mid-stage embryogenesis (e.g. ~100-cell stage) and to the nucleus in oocytes and early embryos ( Fig. 1C -D). The cytoplasmic granules and the nuclear localization of NRDE-3 independent of ERGO-dependent 22G-RNAs will be discussed in more detail in another study (Chen and Phillips 2024) . However, here we want to emphasize that, in an *rde-3* mutant, the GFP:: NRDE-3 (bombardment) strain is expressed and shows the same pattern as GFP::NRDE-3 (CRISPR) in both germline and early-mid embryos ( Fig. 1C -D).

Together, these data indicated that endogenous NRDE-3 is expressed in late-stage germ cells and early embryos, and that the GFP::NRDE-3 construct generated by bombardment is likely a multi-copy transgene that is recognized and silenced by the RNAi pathway. It is also curious to note that NRDE-3 is on the X chromosome, and while the X chromosomes are not thought to be transcriptionally competent through much of the germline, activating chromatin marks are found to be associated with the X chromosome beginning in late pachytene through diplotene and into oocytes (Kelly et al. 2002) . Thus, the expression of NRDE-3 beginning in late pachytene is consistent with other X-linked, oogenic genes that become transcriptionally competent only in late prophase. Lastly, it is worth noting that previous reports using the GFP::NRDE-3 (bombardment) strain and other strains generated by multi-copy transgene insertion methods may need to be reconsidered in the light of transgene silencing potentially confounding germline expression.

## Methods

**Microscopy**

Worms were grown at 20°C according to standard conditions (Brenner 1974) . To obtain early- and mid-stage embryos (8-cell stage and 100-cell stage), gravid adult *C. elegans* were dissected in 12 µL M9 buffer to release embryos. To obtain late-stage embryos (comma stage), embryos laid on plates were picked and transferred to 12 µL M9 buffer. To obtain synchronized young adults, L4s were manually picked and cultured at 20°C for approximately 24 hours. One-day young adult animals were transferred in 12 µL M9 buffer (containing sodium azide). To obtain synchronized L1s, embryos were transferred to unseeded plates and cultured at 20°C for about 24 hours. L1 animals were transferred in 12 µL M9 buffer (containing sodium azide). Embryos and worms in M9 buffer were mounted on a fresh 2% agarose pad for live imaging. At least 10 individual embryos/larvae/adults were imaged for each stage. All images were acquired with a DeltaVision Elite (GE Healthcare) microscope using a 60x N.A. 1.42 oil-immersion objective. A single Z stack was pseusdo-colored using Adobe Photoshop.

**Strain Construction:**

USC1499 *rde-3( ne298 ) I; nrde-3 (tor131[GFP::3xFLAG::nrde-3]) X* , was created by crossing WM30 *rde-3( ne298 ) I* and JMC237 *nrde-3 (tor131[GFP::3xFLAG::nrde-3]) X* . USC1655 *rde-3( ne298 ) I; ggIs1 [GFP::nrde-3] V* was created by crossing WM30 *rde-3( ne298 ) I* and YY174 *ggIs1 [GFP::nrde-3] V* . All strains are available upon request.

## Reagents

***C. elegans* strains:**

**Table:** 

Strain Name	Genotype	Source
YY174	*ggIs1 [GFP:: nrde-3 ] V *	CGC
JMC237	*nrde-3 (tor131[GFP::3xFLAG::nrde-3]) X *	Claycomb lab
USC1499	* rde-3( ne298 ) I; nrde-3 (tor131[GFP::3xFLAG::nrde-3]) X *	this work
USC1655	* rde-3( ne298 ) I; ggIs1 [GFP::nrde-3] V *	this work
